# The Clathrin Assembly Protein PICALM Is Required for Erythroid Maturation and Transferrin Internalization in Mice

**DOI:** 10.1371/journal.pone.0031854

**Published:** 2012-02-21

**Authors:** Mai Suzuki, Hirokazu Tanaka, Akira Tanimura, Kenji Tanabe, Natsuko Oe, Shinya Rai, Syunsuke Kon, Manabu Fukumoto, Kohji Takei, Takaya Abe, Itaru Matsumura, Yuzuru Kanakura, Toshio Watanabe

**Affiliations:** 1 Department of Biological Science, Graduate School of Humanities and Sciences, Nara Women's University, Nara, Japan; 2 Division of Hematology, Department of Internal Medicine, Kinki University, Osaka, Japan; 3 Department of Hematology and Oncology, Osaka University Graduate School of Medicine, Osaka, Japan; 4 Department of Neuroscience, Okayama University Graduate School of Medicine, Dentistry, and Pharmaceutical Sciences, Okayama, Japan; 5 Department of Molecular Immunology, Institute of Development, Aging and Cancer, Tohoku University, Sendai, Japan; 6 Department of Pathology, Institute of Development, Aging and Cancer, Tohoku University, Sendai, Japan; 7 Laboratory for Animal Resources and Genetic Engineering, RIKEN Center for Developmental Biology, Kobe, Japan; Feinberg Cardiovascular Research Institute, Northwestern University, United States of America

## Abstract

Phosphatidylinositol binding clathrin assembly protein (PICALM), also known as clathrin assembly lymphoid myeloid leukemia protein (CALM), was originally isolated as part of the fusion gene *CALM/AF10*, which results from the chromosomal translocation t(10;11)(p13;q14). CALM is sufficient to drive clathrin assembly *in vitro* on lipid monolayers and regulates clathrin-coated budding and the size and shape of the vesicles at the plasma membrane. However, the physiological role of CALM has yet to be elucidated. Here, the role of CALM *in vivo* was investigated using *CALM*-deficient mice. *CALM*-deficient mice exhibited retarded growth *in utero* and were dwarfed throughout their shortened life-spans. Moreover, *CALM*-deficient mice suffered from severe anemia, and the maturation and iron content in erythroid precursors were severely impaired. *CALM*-deficient erythroid cells and embryonic fibroblasts exhibited impaired clathrin-mediated endocytosis of transferrin. These results indicate that CALM is required for erythroid maturation and transferrin internalization in mice.

## Introduction

Clathrin-coated vesicles mediate endocytosis of plasma membrane receptors, channels, and transporters, as well as transmembrane proteins and various soluble macromolecules. As the main component of clathrin-coated pits and vesicles, clathrin forms structures termed triskelia, each composed of three heavy and three light chains [1]–[5], which in turn assemble into a lattice-like structure on the surface of coated pits. The major proteins that drive coated pit formation are the adaptor protein complex AP-2 [1], [6], [7] and clathrin assembly proteins such as phosphatidylinositol-binding clathrin assembly protein (PICALM), also known as clathrin assembly lymphoid myeloid leukemia protein (CALM). The neuronal homolog of CALM, AP180, has been shown to be sufficient for clathrin lattice assembly on lipid monolayers and for the regulation of clathrin-coated buds and the size and shape of vesicles at the plasma membrane [8]–[11].

CALM possesses an AP180 N-terminal homology (ANTH) domain that binds to membrane lipids [9], [12]–[15] and specific motifs that bind to clathrin and AP-2, which are the primary components of clathrin-coated vesicles [11], [14], [15]. Cellular depletion of CALM by RNA interference results in the formation of clathrin-coated structures of abnormal size and shape, which suggests that CALM regulates the proper formation of clathrin-coated vesicles [11].

CALM was originally isolated as a fusion gene, *CALM/AF10*, which results from the chromosomal translocation t(10;11)(p13;q14). This translocation is a cytogenetic abnormality found in acute lymphoblastic leukemia, acute myeloid leukemia and in malignant lymphomas [16]. In a murine bone marrow transplantation model, expression of *CALM/AF10* in primary murine bone marrow cells results in the development of an aggressive form of leukemia [17], [18]. These data suggest that CALM may play an important role in the growth and differentiation of hematopoietic cells. This notion is supported by reports that *fit1* mutants, which contain nonsense point mutations in the *CALM* gene, are anemic, display numerous peripheral blood defects, and are deficient in early hematopoietic progenitor cell populations [19]–[23]. Detailed analysis of the hematopoietic defects of *fit1* mutants has yet to be reported, and so the physiological role of CALM remains elusive.

Using a genetic approach to elucidate the function of CALM in mammals, we established *CALM*-deficient mice through gene targeting. *CALM*-deficient mice were growth retarded *in utero* and remained dwarfed throughout their shortened life-span. In addition, *CALM*-deficient mice suffered from severe anemia due to ineffective erythropoiesis in the bone marrow. These phenotypes resembled those of *fit1* mutants. Moreover, the maturation and iron content of erythroid precursors were severely impaired in *CALM*-deficient mice. In addition, erythroid cells and murine embryonic fibroblasts (MEFs) isolated from *CALM*-deficient mice exhibited impaired clathrin-mediated internalization of transferrin. These data collectively demonstrate that CALM is required for erythroid maturation and transferrin internalization in mice.

## Materials and Methods

### Ethics Statement

This study received specific approval from the Committee of Animal Experiments, Nara Women's University (approval ID 06-13).

### Generation of *CALM*-deficient mice

A genomic clone encompassing the exon that contained the initiation codon (methionine) of *CALM* was isolated from a C57BL/6 BAC library (BACPAC) using Red/ET methods. A targeting vector was constructed in which the marker gene PGK-neo-pA was inserted into this exon. A DT-A fragment was ligated to the 5′ end of the targeting vector for negative selection. The targeting vector was linearized by *Sal* I digestion and introduced into TT2 embryonic stem (ES) cells by electroporation [24]. Of 96 G418-resistant clones, 22 were positive for homologous recombination, as determined by PCR (http://www.cdb.riken.jp/arg/Methods.html), eight clones had undergone homologous recombination as determined by Southern blot analysis. Three clones were injected into 8-cell stage embryos. Chimeras were mated with C57BL/6J females and germline transmission of the disrupted *CALM* allele was confirmed. Heterozygous F1 mice were intercrossed to produce homozygous *CALM*-deficient mice (Acc. No. CDB0683K: http://www.cdb.riken.jp/arg/mutant%20mice%20list.html). Genotyping was carried out using PCR and specific primers designed to amplify either the mutant or wild-type allele. The sequences of the primers used for PCR analysis were as follows: primer A: 5′-ATGTCTGGCCAGAGCCTGACGGACCGAATC-3′ and calm typing C: 5′-GGGTCGGGAGAGGATGCGGGGGGTCTTCAC-3′ for the wild-type allele; and Neogt-1: 5′-CTGACCGCTTCCTCGTGCTTTACG-3′ and calm typing C for the knockout allele.

### Antibodies

For analysis by fluorescence-activated cell sorting (FACS), the following antibodies were purchased from BD Pharmingen: anti-c-Kit (2B8), -Sca-1 (D7), -TER119 (specific for erythroid lineage cells), -Mac-1 (M1/70), -Gr-1 (RB6-8C5), -CD11c (HL3), -B220 (RA3-6B2), -CD44 (IM7), and -CD3e (145-2C11). For immunohistochemistry, an anti-transferrin receptor antibody was purchased from Zymed. Alexa488-conjugated anti-mouse IgG was obtained from Invitrogen. For immunoblot analysis, an anti-β-actin antibody was purchased from Sigma, the anti-CALM polyclonal antibody (G-17) was purchased from Santa Cruz, and the horseradish peroxidase (HRP)-conjugated anti-mouse IgG and anti-goat IgG antibodies were purchased from Thermo Fisher Scientific.

### Immunoblot Analysis

Immunoblot analysis was performed as described previously [25]. Briefly, cells were lysed in SDS-PAGE sample buffer, separated by SDS-PAGE, and then transferred to a nitrocellulose membrane. The membranes were incubated in blocking buffer (140 mM NaCl, 1 mM EDTA, and 20 mM Tris-HCl, pH 7.4) containing 5% bovine serum albumin and 0.02% Tween 20, followed by incubation for 1 h at room temperature with primary antibody. Membranes were incubated with the appropriate secondary antibodies diluted in blocking buffer, and then immunoreactive proteins were visualized by enhanced chemiluminescence (GE Healthcare).

### Flow cytometry

Flow cytometry were performed using a BD FACS Canto II system (BD Biosciences). The data were analyzed using BD FACSDiva software (BD Biosciences) or FlowJo software (TreeStar, Ashland, OR).

### Purification of murine Lin^−^ Sca-1^+^ c-Kit^+^ (LSK) cells

Murine fetal liver (FL) cells were harvested from embryonic day 14.5 (E14.5) embryos and mononuclear cells (MNCs) were obtained by density gradient centrifugation. MNCs were incubated with a cocktail of anti-lineage antibodies (Abs): biotinylated anti-CD3e (145–2C11), -CD45R/B220 (RA3–6B2), -Gr-1 (RB6–8C5), and -TER-119 (TER-119) Abs; fluorescein isothiocyanate (FITC)-conjugated anti-Sca-1 (D7); allophycocyanin (APC)-conjugated anti-c-Kit (2B8); and streptavidin-PE-cy7 (BD Biosciences). LSK cells were obtained by FACS using FACS Aria (BD Biosciences). Staining with 7-amino-actinomycin D (Calbiochem) was used to eliminate non-viable cells.

### Hematopoietic stem cell (HSC) transplantation

LSK cells (1×10^6^) obtained from the fetal livers of *CALM*-deficient and wild-type E14.5 embryos (CD45.2) were transferred into irradiated (1.300 rad) recipients along with 1×10^5^ wild-type LSK cells (CD45.1). The relative contributions from the transferred HSCs were analyzed 9 weeks after transplantation.

### Analysis of intracellular labile iron pool (LIP)

Cellular LIP was measured using the fluorescent metalosensor calcein-AM(CA-AM; Invitrogen), as previously reported, with some modifications [26], [27]. Briefly, cells were incubated for 1 h with or without 500 µM deferoxamine (DFO) (Sigma) in serum-free media (α-MEM). Following washing with phosphate-buffered saline (PBS), cells were incubated with 250 nM CA-AM for 10 min at 37°C in PBS, washed twice, and then resuspended at a density of 1×10^6^/mL in PBS containing 1% bovine serum albumin at room temperature. In addition to CA-AM, as indicated, cells were also incubated with APC-conjugated anti-TER119 and PE-conjugated anti-CD71 Abs. Fluorescence intensity was measured using FACS Canto II (BD Biosciences) in continuous mode. LIP was calculated as the difference in CA mean fluorescence intensity between DFO-treated and -untreated cells.

### Image analysis

Microscopic images of May-Grunwald-Giemsa-stained peripheral smears were obtained using an Olympus (BX51) microscope equipped with a digital camera (Olympus DP71) and processed with DP Controller software.

### *CALM*-deficient embryonic fibroblastoid cell lines

Primary MEFs were generated from mated wild-type or *CALM*-deficient mice 14.5 days after conception. Primary MEFs were immortalized by transfection with a plasmid containing SV40 genomic DNA. Briefly, primary MEFs were plated in six-well plates and transfected with 1 µg of total DNA using Lipofectamine 2000 Reagent (Invitrogen, CA), according to the manufacturer's instructions. Stable immortalized cell clones were obtained by serial dilution. The expression of CALM protein was assessed by immunoblot. MEFs were cultured in Dulbecco's modified Eagle's medium (DMEM) supplemented with 10% fetal calf serum (FCS) and antibiotics (penicillin/streptomycin) at 37°C and 5% CO_2_.

### Internalization assay

To evaluate the uptake of transferrin by erythroblasts, single cell suspensions from fetal liver or bone marrow were incubated in serum-free medium at 37°C for 2 h.

Cells were labeled with 50 µg/ml Alexa Fluor 647-conjugated human transferrin (Invtrogen) in binding buffer (RPMI1640 containing 20 mM HEPES pH 7.4, 1% BSA) on ice for 30 min. After washing to remove unbound transferrin, internalization of transferrin was induced by incubating the cells in buffer (RPMI1640 containing 10% fetal bovine serum) at 37°C for various times. Any transferrin that remained bound to the plasma membrane was removed by incubating the cells in pre-chilled acidic buffer (20 mM MES pH 5, 130 mM NaCl, 50 µM deforoxamine, 2 mM CaCl_2_ and 0.1% BSA) on ice for 20 min. After washing three times, cells were labeled with FITC-conjugated anti-TER119 Ab and the fluorescence intensity of internalized transferrin in the erythroblast cell population (TER119-high cells) was quantified by FACS Canto II (BD Biosciences).

To detect the internalization of transferrin in MEFs, cells on coverslips were incubated with 10 µg/ml Alexa Fluor 488-conjugated human transferrin (Invitrogen) for 20 min at 37°C, and then fixed with 3.7% formaldehyde in PBS for 15 min at room temperature. After washing with PBS, cover slips were mounted on glass slides using Prolong Gold (Invitrogen) and then analyzed by spinning disc confocal microscopy (CSU10; Yokogawa Electric Co.) using an inverted microscope (IX-71; Olympus) equipped with an Ar/Kr laser, as described previously [25]. To quantitate the internalization of transferrin in MEFs, the fluorescence intensity of randomly selected individual cells (total cell area) was measured and processed using Image J software (http://rsbweb.nih.gov/ij/index.html).

## Results

### Generation of *CALM*-deficient mice

**Figure 1:**
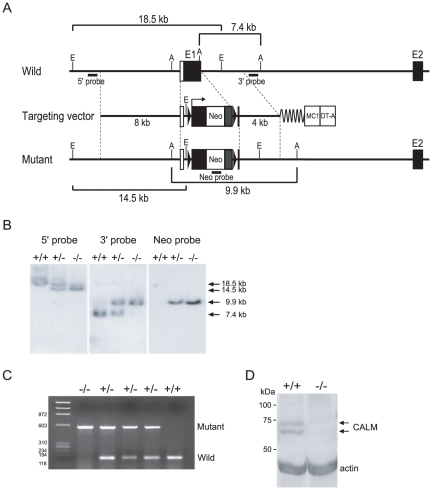
Targeted disruption of the *CALM* gene. (A) Schematic of the targeting strategy used for disruption of the first coding exon of *CALM*. (B) Southern hybridization analysis of DNA isolated from targeted mouse embryos. From left to right: 5′ probe, 3′ probe and neo probe, as shown in A. (C) Genomic PCR analysis of DNA isolated from mouse tail. The genotypes are indicated above each lane. (D) Immunoblot analysis of stable MEF cell lines from wild-type (+/+) and *CALM*-deficient (−/−) mouse embryos (E14.5). The expected molecular weights of CALM are 62 and 72 kDa (arrows). Actin was used as a loading control.

To address the functional role of CALM in mice, *CALM*-deficient mice were generated using a gene targeting approach. The *CALM*-targeting construct was designed with an expression cassette containing the neomycin-resistance gene (*neo* ^r^) inserted just after the initiation ATG codon in exon 1 of the *CALM* gene (Fig. 1A). Eight clonal ES cell lines bearing the targeted allele were identified by Southern blot analysis, of which three were used to generate chimeric mice with germ line transmission of the targeted *CALM* allele. Their offspring were used for all studies reported herein. Heterozygous mice exhibited no obvious abnormalities. To generate homozygous *CALM*-deficient mice, F1 heterozygous mice were interbred and the F2 offspring were genotyped by PCR and Southern blot (Fig. 1B and C). The absence of CALM protein expression in *CALM*-deficient mice was confirmed by immunoblot (Fig. 1D).

### *CALM*-deficient mice exhibit significant growth defects and a shortened life-span

**Figure 2:**
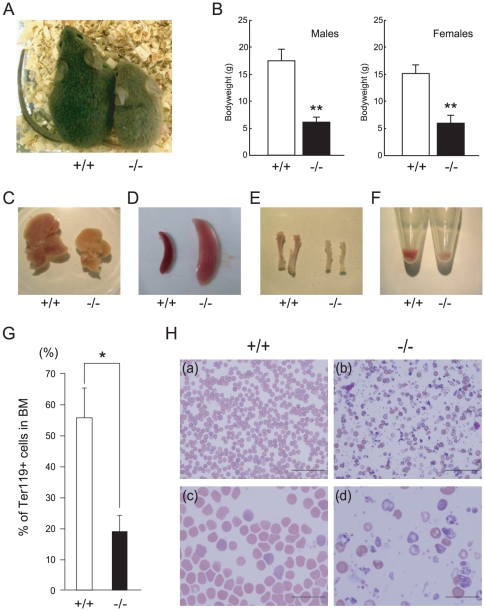
Growth retardation and peripheral blood morphology in postnatal *CALM*-deficient mice. (A) Wild-type (+/+) (left) and *CALM*-deficient (−/−) (right) mice at 3 weeks of age. (B) Average weight of wild-type (+/+) and *CALM*-deficient (−/−) mice at postnatal day 28. Data shown represent *CALM*-deficient males (n = 3), *CALM*-deficient females (n = 4), wild-type males (n = 36), and wild-type females (n = 35). **, *P*<0.01 vs control. Liver (C), spleen (D) and tibia (E) from wild-type (+/+) or *CALM*-deficient (−/−) mice at 3 weeks of age. (F) Bone marrow cells were collected by centrifugation. Note that the pellet from *CALM*-deficient (−/−) mice is small and pale in color. (G) Quantification of TER119-positive bone marrow cells (data represent means±SD; n = 5). *, *P*<0.05 vs. control. (H) Peripheral blood smears from wild-type (+/+) littermates (left panels, a and c) or *CALM*-deficient (−/−) mice (right panels, b and d) at 3 weeks of age. Magnification: upper panels, 400×; lower panels, 1000×. Scale bar, 50 µm (upper panel) and 20 µm (lower panel).

*CALM*-deficient mice were obtained at the expected Mendelian ratios (Table 1). The *CALM*-deficient mice were smaller in size compared to their wild-type littermates (Fig. 2A), and more than 90% died between birth and weaning (Table 1). To characterize the retarded growth phenotype of *CALM*-deficient mice in more detail, the body weight of *CALM*-deficient mice and their wild-type littermates was assessed. As shown in Figure 2B, *CALM*-deficient mice weighed, on average, 35.3% (for males) and 39.7% (for females) of the weight of normal littermates at weaning (28 days of age). Because *CALM*-deficient mice exhibited a growth retardation phenotype at birth, we also assessed whether *CALM* was necessary for normal prenatal growth. The weight of *CALM*-deficient embryos was compared to that of their wild-type littermates at E14.5. *CALM*-deficient embryos were approximately 74% of the size of normal littermates (data not shown), which suggested that *CALM* is also required for normal prenatal growth. The phenotype of *CALM*-deficient mice therefore was retarded growth *in utero* with mice remaining dwarfed throughout their life-span.

**Table 1:** Genotypes of intercrossed CALM^+/−^ mice.

Stage	+/+	+/−	−/−
P28	72 (69)	138	8 (69)
P0	23 (25)	50	8 (25)
E19.5	9 (9)	17	6 (9)
E18.5	16 (13)	26	12 (13)
E14.5	17 (15)	29	18 (15)

The total number of embryos or postnatal mice isolated for each genotype is indicated, with values in parentheses indicating the number of embryos or postnatal mice expected based on Mendelian distribution.

It should be noted that after back-crossing five times with C57B6/J mice, no *CALM*-deficient mice were obtained, which suggested that *CALM* deficiency may result in a lethal phenotype that is dependent on the genetic background of B6 and CBA mice.

### *CALM*-deficient mice suffer from severe anemia

Many of the tissues in *CALM*-deficient mice, including liver, kidney and tibia, were smaller compared to wild-type mice (Fig. 2C, E). By marked contrast, however, the spleens of *CALM*-deficient mice were larger than those of their wild-type littermates, which was indicative of splenomegaly (Fig. 2D). Histologic examination revealed that follicles were absent in the spleens of *CALM*-deficient mice, which indicating a defect in the B cell population. This was confirmed by FACS analysis, which was consistent with impaired B cell maturation in the spleens of *CALM*-deficient mice (data not shown). In addition, there were fewer bone marrow cells in *CALM*-deficient mice and the isolated cell pellets were pale in appearance (Fig. 2F), which suggested a defect in the regulation of hematopoiesis. In fact, the number of TER119-positive erythroid cells in the bone marrow of *CALM*-deficient mice was significantly reduced compared to control littermates (Fig. 2G). We also observed a ballooning and granular degeneration of hepatocytes (data not shown), indicative of hepatocyte damage. As shown in Table 2, the number of red blood cells (RBCs) in *CALM*-deficient mice was significantly reduced compared to control littermates, and the mice were severely anemic, with dramatically lower hemoglobin levels. Additionally, peripheral blood in *CALM*-deficient mice contained hypochromic RBCs (Fig. 2H).

**Table 2:** Hematopoietic parameters of control and CALM^−/−^ mice.

Genotype	WBC	RBC	Hb	HCT	MCV	MCH	MCHC	PLT
+/+	3.7±0.98	875.3±65.5	13.2±0.85	47.4±3.29	54.1±0.44	15.0±0.18	27.8±0.17	80.0±54.45
+/−	2.3±0.49	840.0±52.4	12.6±0.88	46.5±2.42	55.4±2.13	15.0±0.97	27.1±0.74	103.0±29.90
−/−	3.4±0.90	267.8±97.0	3.7±1.29	14.2±4.71	53.4±4.80	13.9±1.17	26.1±1.01	118.6±23.34

Data were obtained from 3-week-old mice. WBC, white blood cell count (×10^3^/µl); RBC, red blood cell (×10^4^/µl); Hb, hemoglobin (g/dl); HCT, hematocrit (%); MCV, mean corpuscular volume (fl); MCH, mean corpuscular hemoglobin (pg); MCHC, mean corpuscular hemoglobin concentration (g/dl); Plt, platelet count (×10^4^/µl). Data are the mean ± SD of four wild-type, four CALM^+/−^, and five CALM^−/−^ mice. Statistical analysis was carried out using the unpaired t-test. **P<0.01, *P<0.05.

### Maturation of erythroid cells is impaired in *CALM*-deficient mice

**Figure 3:**
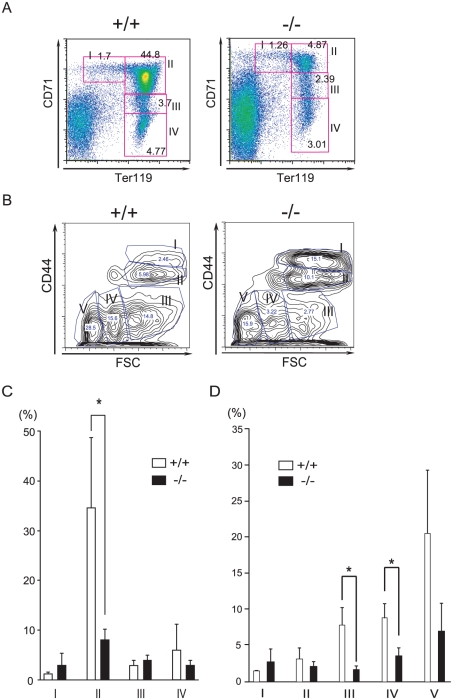
FACS analyses of erythroblasts at different stages of maturation in wild-type or *CALM*-deficient mice. (A) Representative flow cytometry histograms. In 3-week-old mice, erythroblasts at different maturation stages were identified by double staining with FITC-conjugated anti-TER119 and PE-conjugated anti-CD71 Abs. (B) Representative flow cytometry histograms. Erythroblasts at different maturation stages were identified by double staining with FITC-conjugated anti-TER119 and APC-conjugated anti-CD44 Abs. Plots of CD44 vs. forward scatter (FSC) for TRE119-positive cells are shown. (C) Quantification of regions I to IV of (A) (data represent means±SD; n = 4). *, *P*<0.05 vs. control. (D) Quantification of regions I to V of (B) (data represent means±SD; n = 5). *, *P*<0.05 vs. control.

To investigate whether the severe anemia in *CALM*-deficient mice was due to defects in erythroid differentiation, the expression of transferrin receptor 1 (CD71) and TER119 was analyzed by FACS, which allows the different stages of maturation of murine erythroblasts to be distinguished [28]. Bone marrow cells were immunostained with FITC-conjugated anti-TER119 and PE-conjugated CD71 Abs (Fig. 3A, C). A representative FACS histogram is shown in Figure 3A. Region I of the histogram (CD71^high^TER119^low^) represents the proerythroblast population or immature erythroblasts. Region II (CD71^high^TER119^high^) represents the basophilic erythroblast population. Region III (CD71^low-med^TER119^high^) represents the polychromatic and orthochromatic erythroblast populations, which are hemoglobin-producing cells, and region IV (CD71^low^TER119^high^) represents mature erythrocytes. This profile is known as Socolovsky's plot. As shown in Figure 3A and C, *CALM*-deficient mice had significantly fewer cells in region II compared with their wild-type littermates. These results suggested that erythroid maturation at the stage of proerythroblast to immature erythroblast is impaired in *CALM*-deficient mice.

To confirm these results, we also analyzed the expression levels of CD44 and TER119, the relative levels of which have been shown to correlate with different maturation stages of murine erythroblasts [29]. Bone marrow cells were subjected to immunostaining with FITC-conjugated anti-TER119 and APC-conjugated CD44 Abs (Fig. 3 B, D). As shown in Figure 3B and D, *CALM*-deficient mice had significantly lower numbers of cells in region III compared with their wild-type littermates, which supported the idea that erythroid maturation from proerythroblast to immature erythroblast is impaired in *CALM*-deficient mice.

These data collectively suggested that the maturation of erythroid cells is severely impaired in *CALM*-deficient mice.

### The erythroid defects in *CALM*-deficient mice are cell autonomous

**Figure 4:**
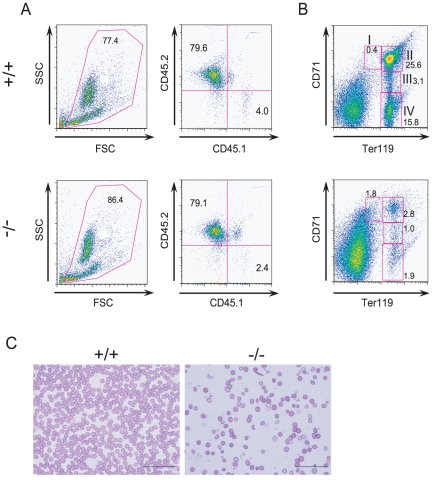
*CALM* is required for normal erythropoiesis. (A) CD45 profile of peripheral blood cells from transplanted mice 9 weeks after transplantation. (B) FACS analyses of erythroblasts at different maturation stages from transplanted mice using anti-TER119 and anti-CD71 Abs. (C) Peripheral blood smears from mice transplanted with fetal liver cells from either wild-type littermates or *CALM*-deficient embryos. Scale bar, 50 µm. In A, B, and C, (+/+) and (−/−) represent host mice transplanted with cells from wild-type and *CALM*-deficient mice, respectively.

HSC activity was analyzed by transplantation assay. Fetal liver cells from wild-type or *CALM*-deficient embryos (CD45.2) were transplanted into X-ray irradiated host mice along with a one-tenth fraction of wild-type cells (CD45.1) as a competitor. After 9 weeks, CD45.2-positive hematopoietic cells derived from *CALM*-deficient embryos had repopulated the bone marrow at the same rate as wild-type cells (Fig. 4A). However, after 12 weeks, impaired maturation of early to late stage erythroblasts and abnormal erythroid cells were observed only in host mice transplanted with fetal liver cells from *CALM*-deficient embryos (Fig. 4B and C). The impaired maturation of erythroid cells was confirmed by anti-CD44 staining (data not shown).

In addition, host mice transplanted with fetal liver cells from *CALM*-deficient embryos were anemic and had fewer RBCs and lower hemoglobin levels than wild-type transplanted mice (data not shown). These results indicated that the observed erythroid defects in *CALM*-deficient mice were cell autonomous and not a result of the hematopoietic environment.

It should be noted that host mice transplanted with fetal liver cells from *CALM*-deficient embryos also exhibited splenomegaly, and that there was a similar pattern of progenitor activity in spleen cells as in bone marrow (data not shown). On the other hand, no progenitor activity was detected in spleen cells from host mice transplanted with fetal liver cells from wild-type embryos.

### Incorporation of iron is reduced in erythroid cells of *CALM*-deficient mice

Erythroid cells from *CALM*-deficient mice were severely anemic and erythroid maturation was impaired (Fig. 2, 3 and 4). Since iron deficiency is known to inhibit erythroid differentiation and induce anemia [30], [31], we investigated whether an iron-deficiency in erythroid cells in *CALM*-deficient mice was the cause of the anemia.

**Figure 5:**
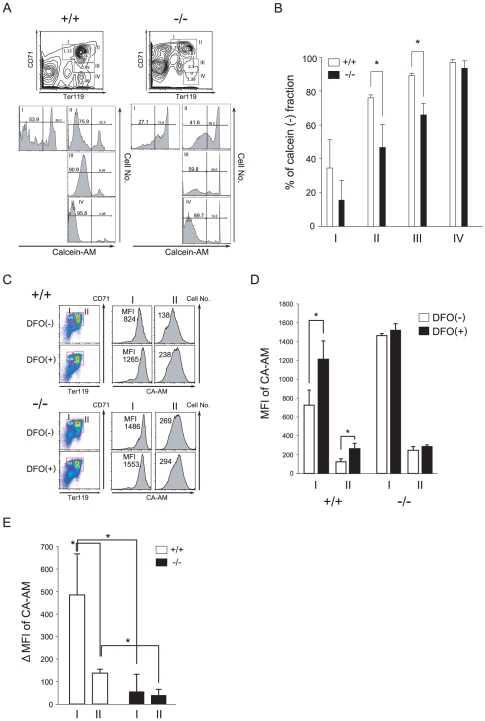
Flow cytometry analysis of the LIP. (A) Representative flow cytometry histograms. Erythroblasts at different stages of maturation were identified by double staining with APC-conjugated anti-TER119 and PE-conjugated anti-CD71 Abs (upper). These cells were simultaneously loaded with calcein (CA-AM) for the LIP assay (lower). (B) Quantification of CA-negative fractions in regions I to IV of (A) (data represent means±SD; n = 3). (C) Representative flow cytometry histograms of E14.5 fetal liver cells. Erythroblasts at different stages of maturation were identified by double staining with APC-conjugated anti-TER119 and PE-conjugated anti-CD71 Abs. Cells were treated for 1 h with the iron chelator DFO, loaded with CA-AM for 10 min, and then analyzed. (D) Representative fluorescence (FL1-high) histograms of CA-stained cells treated with or without DFO are shown, along with mean fluorescence intensity (MFI). (E) The LIP is represented by the difference in MFI between DFO-treated and -untreated cells (data represent means±SD; n = 3). *, *P*<0.05 vs. control. In A, B, C and D, (+/+) and (−/−) represent wild-type and *CALM*-deficient mice, respectively.

Iron bound to transferrin is taken up by cells via transferrin receptor 1-mediated endocytosis. This is the main pathway of iron intake in erythroid cells. We first analyzed amount of ferritin and transferrin in the plasma of wild-type and *CALM*-deficient mice by ELISA (ICL, Portland, OR, USA). There were no differences in the levels of transferrin in wild-type and *CALM*-deficient mice, whereas ferritin levels, which directly correlate with the total amount of iron stored in the body, were three-times higher in *CALM*-deficient mice than wild-type mice. These results suggested that there was an excess of iron in the body of *CALM*-deficient mice, despite the hypochromic and microcytic anemic phenotype. This suggested that there was an imbalance in the distribution of iron from erythroid cells to other tissues in *CALM*-deficient mice. To investigate this possibility, we examined the labile (or chelatable) iron pool (LIP) in erythroid cells, which is a point of convergence of the iron metabolic pathways. The LIP was measured using the cell permeable iron chelator CA-AM, as previously described [26], [27]. Upon uptake by viable cells, CA-AM undergoes hydrolysis by esterases to form calcein (CA), the fluorescence of which can be measured (excitation wavelength of 488 nm, emission wavelength of 517 nm). CA fluorescence is quenched upon binding to the cellular LIP in a stoichiometric fashion. The presence of a high-affinity chelator such as desferoxamine (DFO) results in extraction of iron from CA-iron complexes and a corresponding increase in CA fluorescence. Thus, the difference in cellular fluorescence in the presence and absence of DFO can be taken as a measure of the LIP. The LIP is dependent on cell type and maturation stage; analysis of erythroid cells in the blood, bone marrow and in culture has shown that the LIP decreases during maturation [26], [27]. Compared to wild-type cells, erythroid cells in the bone marrow of *CALM*-deficient mice exhibited higher CA fluorescence intensity in regions I through III of the Socolovsky plot (Fig. 5A, B). As shown in Fig. 5C and D, the mean fluorescence intensity (MFI) of CA-AM-loaded wild-type fetal liver erythroid cells at stages I and II was 762 and 126, respectively, and MFI was significantly increased to 1211 and 263, respectively, by treatment with DFO. Based on the difference in MFI in DFO-treated and -untreated cells, the LIP in wild-type cells in stages I and II was 484±181 and 137±17 (MFI±S.D; n = 7), respectively (Fig. 5E). On the other hand, in *CALM*-deficient cells, there was only a small increase in CA fluorescence in the presence of DFO, which suggested that the cellular LIP in *CALM*-deficient cells was smaller than in wild-type cells. The LIP also appeared to be relatively constant in *CALM*-deficient cells (55±76 and 39±26 at stages I and II, respectively; n = 3). Similar results were obtained using bone marrow cells from host mice transplanted with fetal liver cells from wild-type or *CALM*-deficient embryos (data not shown).

Several reports show that CA is pumped out of cells via the multidrug resistance machinery [32]. There were no apparent differences in mean CA-fluorescence intensity in DFO-treated wild-type and *CALM*-deficient cells, which indicated that there were similar levels of CA in both cell types (Fig. 5 D). Thus, it is unlikely that the differences in LIP were due to the multidrug resistance machinery. Taken together, these results indicated that the iron content of erythroid cells at stages I and II is lower in *CALM*-deficient mice compared to their wild-type littermates. Therefore, altered distribution of iron in *CALM*-deficient mice inhibits erythroid maturation and results in anemia.

### Reduced incorporation of transferrin into erythroid cells and MEFs derived from *CALM*-deficient mice

Transferrin receptor 1, also known as CD71, is highly expressed in erythroblasts, and uptake of iron-bound transferrin through transferrin receptor 1 is the main pathway of iron uptake in erythroid precursors [33], [34]. It is also well established that inhibition of this process inhibits erythroid maturation. Because transferrin is internalized via clathrin-dependent endocytosis [6], [35], and CALM localizes to clathrin-coated pits, clathrin-mediated endocytosis of transferrin in *CALM*-deficient mice was assessed [11], [15].

**Figure 6:**
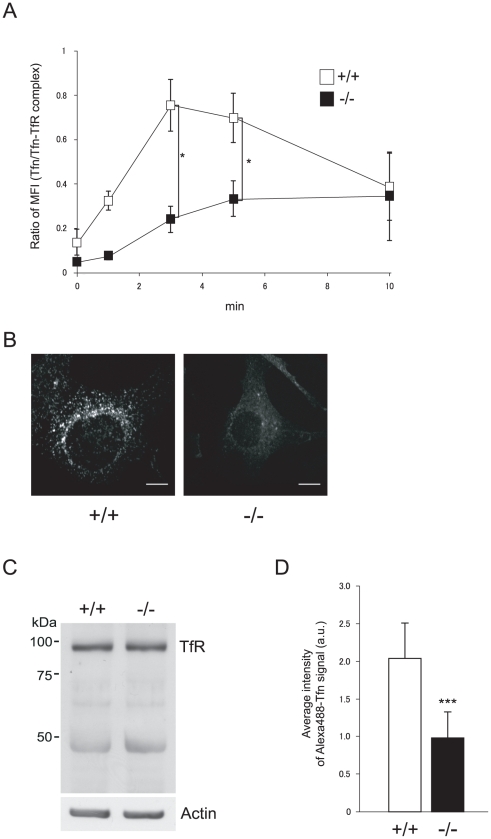
Impaired transferrin internalization in *CALM*-deficient erythroid cells and MEFs. (A) Uptake of transferrin in wild-type or *CALM*-deficient erythroid cells. Single cell suspensions from fetal livers were analyzed as described in Materials and Methods. Transferrin uptake was normalized to the ratio of mean anti-CD71 (transferrin receptor 1) fluorescence intensity between wild-type and *CALM*-deficient cells to control for differences in transferrin receptor expression. (B) Uptake of transferrin by wild-type or *CALM*-deficient MEFs was analyzed as described previously [40]. Scale bar, 10 µm. (C) Expression of transferrin receptor 1 in wild-type and *CALM*-deficient MEFs was analyzed by immunoblot. Actin was used as a loading control. (D) Quantification of transferrin uptake was performed as described in Materials and Methods (data represent means±SD; n = 50 for wild-type cells and n = 54 for *CALM*-deficient cells). ***, *P*<0.001 vs. control. Fluorescence is shown in arbitrary units (*a.u.*). In A, B, C and D, (+/+) and (−/−) represent wild-type and *CALM*-deficient MEFs, respectively.

Transferrin uptake by erythroid cells from fetal liver was assessed by FACS, as described in Materials and Methods. The level of transferrin receptor (CD71) expression in TER119-positive erythroid cells from *CALM*-deficient mice was about 2.5-fold higher than that seen in wild-type cells (3365±1398 and 8554±1681 MFI±S.D, respectively; n = 3), which could reflect differences in the internalization of transferrin receptors. Thus, transferrin uptake was normalized to transferrin receptor level for this analysis. In wild-type cells, 62% of bound transferrin was rapidly internalized after 3 min. On the other hand, only 19% of bound transferrin was internalized by *CALM*-deficient cells (Fig. 6A). Similar results were obtained using wild-type and *CALM*-deficient erythroid cells derived from neonatal bone marrow (data not shown).

To examine this defect in transferrin internalization in more detail, MEFs were established from wild-type and *CALM*-deficient E14.5 embryos. Wild-type MEFs expressed the transferrin receptor and transferrin was internalized in a constitutive manner (Fig. 6B). However, *CALM* knock-out MEFs derived from *CALM*-deficient embryos failed to incorporate transferrin (Fig. 6B), despite similar overall levels of transferrin receptor (Fig. 6C). Transferrin uptake in wild-type and *CALM*-deficient MEFs was quantitated and the results are shown in Fig. 6D. Uptake of transferrin by wild-type MEFs was approximately twice that seen in *CALM*-deficient cells. These results indicated that the internalization of transferrin receptors is abrogated in *CALM*-deficient cells. Thus, the erythropoietic defects in *CALM*-deficient mice may be due to inhibition of transferrin endocytosis in the absence of CALM.

## Discussion

In this report, we established *CALM*-deficient mice and showed that these mice exhibit retarded growth *in utero* and remain dwarfed throughout their shortened life-span. *CALM*-deficient mice suffer from severe anemia, similar to what has been reported for *fit1* mutant mice [21]. In addition, the maturation of erythroid precursors was severely impaired in mice lacking CALM. The iron content of erythroid precursors was lower in *CALM*-deficient mice compared to their wild-type littermates. Importantly, those phenotypes were reconstituted in transplantation experiments. The amount of ferritin in *CALM*-deficient mice was three-times higher than that in wild-type mice, suggesting that there is an excess of iron in the plasma of mice deficient in CALM.

It is well established that iron deficiency inhibits erythroid maturation [26], [27], [33], [34]. Anemia is the most obvious manifestation of iron deficiency due to the large amount of iron required for hemoglobin production in developing erythroid cells. *Transferrin receptor 1*-deficient mice die during embryogenesis due to the lack of transferrin-mediated iron uptake [31]. Slc11a2 is the only transmembrane transporter protein known to be required for iron entry into cells and is required for normal hemoglobin production during the development of erythroid precursors. *Slc11a2*-deficient mice also exhibit severe anemia after birth [30]. Our results suggest that iron deficiency in *CALM*-deficient mice results in inhibition of erythroid maturation and subsequent anemia.

The question then arises as to which molecule involved in iron uptake is affected by *CALM*-deficiency in mice. Transferrin receptor 1 (TFR1), also known as CD71, is one such candidate molecule. TFR1 is highly expressed in erythroblasts, and the uptake of iron-bound transferrin through TFR1 is the main pathway by which iron enters erythroid precursors [33], [34]. It is also well established that inhibition of this process prevents erythroid maturation due to iron deficiency [31]. Transferrin is taken up by cells through clathrin-dependent endocytosis [6], [35]; thus, it is likely that impaired transferrin endocytosis in *CALM*-deficient mice underlies the inhibition of erythroid maturation. However, the involvement of CALM in clathrin-dependent endocytosis of transferrin remains controversial. While one study has shown that over-expression of full-length CALM and CALM fragments that contain clathrin-binding domains can block transferrin endocytosis [15], another study reports that over-expression of CALM had no effect on transferrin endocytosis [36]. CALM depletion experiments using small inhibitory (si)RNA did not demonstrate an effect on transferrin endocytosis, although there was partial inhibition of epidermal growth factor (EGF) receptor endocytosis [36], [37]. Based on these reports, CALM is not believed to be an essential component of clathrin-associated endocytosis of transferrin [11], [36], [37]. The *CALM*-deficient mice generated in the current study provided an excellent model system for probing the role of CALM in clathrin-dependent endocytosis of transferrin. Transferrin uptake by erythroid cells was analyzed by FACS, and the results suggested that transferrin uptake is significantly attenuated in *CALM*-deficient erythroid cells. We also established *CALM* knock-out (KO) MEFs from *CALM*-deficient embryos and clearly demonstrated that *CALM* KO MEFs fail to incorporate transferrin. Thus, CALM does indeed appear to be involved in transferrin endocytosis in erythroid cells and MEFs. In addition, the LIP in erythroid cells, which represents a crossroads of iron metabolic pathways, was determined. The iron content of erythroid cells was lower in *CALM*-deficient mice compared to their wild-type littermates. Our results collectively suggest that defective transferrin endocytosis in *CALM*-deficient mice results in altered distribution of iron in the body and inhibition of erythroid maturation, resulting in an anemic state.

Discrepancies between the results obtained by RNA interference using cell lines and those derived from genetic models in mice are reported for several proteins involved in clathrin-mediated endocytosis, such as SMAP1, epsin1 and epsin 2 [36]–[42]. In these cases, impaired endocytosis was reported in response to the inhibition of protein expression by siRNA but not in gene-deficient mice. As suggested previously for other proteins, differences between our results and those of previous studies in terms of the involvement of CALM in transferrin endocytosis could be due to incomplete inhibition of protein expression using siRNA [36], [37].

*CALM* was originally isolated as a component of the *CALM/AF10* fusion gene, which results from the chromosomal translocation t(10;11)(p13;q14) [16]. In a murine bone marrow transplantation model, expression of *CALM/AF10* in primary murine bone marrow cells results in the development of an aggressive form of leukemia [17], [18]. These data suggest that CALM or clathrin-mediated endocytosis may play an important role in leukemogenesis. Recent reports demonstrate that the clathrin-binding domain of *CALM* is important for leukemogenesis by *CALM-AF10* [43], [44]. The fact that deletion of *CALM* resulted in disruption of clathrin-mediated endocytosis of transferrin suggests that clathrin-mediated endocytosis may play a role in leukemogenesis. A number of other proteins that are important for hematopoiesis are substrates of clathrin-mediated endocytosis [45]–[47]. Thus, the *CALM*-deficient mice and MEFs established in this study will be useful tools for elucidating the involvement of *CALM* in leukemogenesis.

*CALM* plays an important role in the central nervous system (CNS). For example, reduction of CALM expression in hippocampal neurons results in dendritic dystrophy [48]. Although the current study focused mainly on hematological changes in *CALM*-deficient mice, we did some preliminary analyses of the mouse brain to assess any pathology of the neural system. Although there were no differences observed in the hippocampus and dentate gyrus between wild-type and *CALM*-deficient mice, there was significant atrophy of the cortex and ventricles were markedly enlarged in *CALM* -deficient mice (data not shown). A detailed analysis of these observations will be reported elsewhere.

In summary, our analysis of *CALM*-deficient mice supports an important role for CALM in erythroid maturation and transferrin incorporation via clathrin-dependent endocytosis. A number of other proteins that are important for hematopoiesis are also incorporated into cells via clathrin-mediated endocytosis [45]–[47]; thus, *CALM*-deficient mice and MEFs established from these mice should prove to be useful for studying the involvement of CALM in other important hematopoietic processes. Several such experiments are currently underway in our laboratory.
